# Analysis of bacterial diversity and community structure in gastric juice of patients with advanced gastric cancer

**DOI:** 10.1007/s12672-023-00612-7

**Published:** 2023-01-20

**Authors:** Qiang Wei, Qi Zhang, Yinhang Wu, Shuwen Han, Lei Yin, Jinyu Zhang, Yuhai Gao, Hong Shen, Jing Zhuang, Jian Chu, Jiang Liu, Yunhai Wei

**Affiliations:** 1 Huzhou Central Hospital, Affiliated Central Hospital Huzhou University, No.1558, Sanhuan North Road, Wuxing District, Zhejiang Province 313000 Huzhou, People’s Republic of China; 2 Zhejiang Chinese Medical University, Hangzhou, Zhejiang Province People’s Republic of China; 3 Key Laboratory of Multiomics Research and Clinical Transformation of Digestive Cancer of Huzhou, Huzhou, People’s Republic of China

**Keywords:** Gastric cancer, Advanced gastric cancer, Microbiota, Gastric juice, Screening

## Abstract

**Background:**

The occurrence and development of gastric cancer are related to microorganisms, which can be used as potential biomarkers of gastric cancer.

**Objective:**

To screen the microbiological markers of gastric cancer from the microorganisms of gastric juice.

**Methods:**

Gastric juice samples were collected from 61 healthy people and 78 patients with gastric cancer (48 cases of early gastric cancer and 30 cases of advanced gastric cancer). The bacterial 16 S rRNA V1-V4 region of gastric juice samples was sequenced. The Shannon index, Simpson index, Ace index and Chao index were used to analyze the diversity of gastric juice samples. The RDP classifier Bayesian algorithm was used to analyze the community structure of 97% OTU representative sequences with similar levels. Linear discriminant analysis and ST-test were used to analyze the differences. Six machine learning algorithms, including the logistic regression algorithm, random forest algorithm, neural network algorithm, support vector machine algorithm, Catboost algorithm and gradient lifting tree algorithm, were used to construct risk prediction models for gastric cancer and advanced gastric cancer.

**Results:**

The microbiota diversity and the abundance of bacteria was different in the healthy group, early gastric cancer and advanced gastric cancer (P < 0.05). The top five abundant bacteria among the three groups were *Streptococcus, Rhodococcus, Prevotella, Pseudomonas* and *Helicobacter.* Bacterial flora such as *Streptococcus, Rhodococcus* and *Ochrobactrum* were significantly different between the healthy group and the gastric cancer group. The accuracy of the random forest prediction model is the highest (82.73% correct). The bacteria with the highest predictive value included *Streptococcus, Lactobacillus* and *Ochrobactrum*. The abundance of bacteria such as *Fusobacterium, Capnocytophaga, Atopobium, Corynebacterium* was high in the advanced gastric cancer group.

**Conclusion:**

Gastric juice bacteria can be used as potential biomarkers to predict the occurrence and development of gastric cancer.

**Supplementary Information:**

The online version contains supplementary material available at 10.1007/s12672-023-00612-7

## Introduction

Gastric cancer (GC) is the most common cancer and the second leading cause of cancer death worldwide [1, 2]. According to the latest global cancer data released by the International Agency for Research on Cancer (IARC) of the World Health Organization (WHO), there were approximately 480,000 new cases of GC in China in 2020, accounting for approximately 4% of the global new cases of GC [3]. GC is a disease caused by multiple environmental factors and alternative carcinogenic pathways [4]. Timely radical surgery for early GC can effectively control disease progression and even cure the disease under certain conditions. Some studies have reported that the 5-year survival rate of early GC is over 90% [5]. Due to the lack of ideal tumor markers for early screening and diagnosis, 70% of patients with GC are found to be in an advanced stage, there is little progress in improving the prognosis of patients with advanced GC, and the 5-year overall survival rate is still less than 25% [6]. Most tumor markers used for early screening are based on blood, and their sensitivity is not satisfactory due to the low level of tumor markers in blood [7]. At present, pathological biopsy of gastric mucosa under gastroscopy is still the most reliable method for GC diagnosis. However, pathological examination is invasive, and puncture causes tissue damage. In some tumors with abundant blood supply, sometimes there is more bleeding or even bleeding that is difficult to stop, which easily causes infection. In addition, only diseases with morphological changes can be diagnosed, only pathological changes in a certain stage of the development of a disease can be reflected, and only local lesions can be reflected in the submitted samples. If the samples are not taken properly, missed diagnosis or misdiagnosis can easily result. However, gastric juice only appears in the stomach. Compared with biopsy, gastric juice covers a larger gastric surface area and can directly contact the gastric mucosa to reflect the real state in the stomach more sensitively [8]. Moreover, PCR experiments showed that the method of using gastric juice samples was more advantageous than conventional gastric biopsy samples [6]. Tsuda et al. [9] showed that gastric juice samples had higher diversity than gastric mucosa samples. Compared with gastric juice samples, the components of *Helicobacter pylori (HP)* and proteinobacteria were higher in gastric mucosa samples. Compared with gastric juice samples, *Actinomycetes, Bacteroides* and *Firmicutes* were less common in mucosal samples. However, there was no significant difference between them. Therefore, gastric juice samples can replace gastric mucosa samples and are more convenient to operate in clinical practice.

An increasing number of studies have shown that the gastric microbiota is closely related to the occurrence and development of GC. The microbial communities of the gastric microenvironment are dominated by *Firmicutes, Actinobacteria, Bacteroidetes* and *Proteobacteria*, as well as species of *Lactobacillus, Streptococcus* and *Propionibacillus*. The composition of the gastric microbiota is highly dynamic and influenced by acid inhibition, gastric inflammation and *HP* [10]. GC may be the result of a complex interaction between gastric microbiota and *HP*. Bacteria can enhance carcinogenesis by promoting inflammation, stimulating cell proliferation, changing stem cell dynamics and producing toxic metabolites [11]. Lertpiryapong et al. [12] found that intestinal microbiota (including *Clostridium, Lactobacillus* and *Bacteroides*) colonized the stomach, resulting in an increase in gastric intraepithelial neoplasia to 69% in male mice 7 months after *HP* infection. Eun et al. [13] found that the gastric microbiome of the GC group was more diverse than that of other chronic gastritis and intestinal metaplasia groups, especially *HP*-positive patients. Aviles-Jimenez et al. [14] compared the differences in microbes in the stomach of nonatrophic gastritis, intestinal metaplasia and intestinal GC by gene chip and found that *Porphyromonas, Neisseria* and *Streptococcus* were reduced in the gastric mucosa of GC patients, and *Lactobacillus coleohominis, Lachnospira Bryant* and *Pseudomonas aeruginosa were* increased. These results suggest that these bacteria may play a role in the process from gastritis to intestinal metaplasia to GC. Yu et al. [15], using 16 S ribosomal RNA gene sequencing analysis and the PICRUSt bioinformatics software package, showed that the diversity of bacteria in the stomach may play an important role in the occurrence of gastric cardia cancer. Sung [16] showed that human gastric juice contains different microbial groups mainly composed of *Fusobacteria, Actinomycetes, Bacteroidetes, Firmicutes* and *Proteobacteria*. Therefore, the study of gastric juice microbiota can provide a theoretical basis for the mechanism of GC progression.

The composition of the microbiota in the stomach can be influenced by external factors, such as diet, proton pump inhibitors, antibiotics, etc. In vivo animal experiments have found [17] that long-term use of yogurt containing probiotics such as *Bifidobacterium* and *Lactobacillus* can reduce the inflammatory response and inhibit intestinal metaplasia induced by *HP* infection in Mongolian gerbils, possibly because probiotics can induce an increase in IL-10 expression and a decrease in TNF-α expression. Paroni et al. [18] used 16 S rRNA gene sequencing in patients with dyspepsia and showed a different gastric microbiota between patients treated with and without omeprazole. Rosenvinge et al. [19] described bacterial and fungal microbiota in the gastric juices of 25 patients by gene sequencing of bacterial 16 S rRNA variable regions and fungal ITS regions and concluded that antibiotics can reduce bacterial but not fungal biodiversity. Therefore, actively searching for different gastric juice microbiota in GC provides a basis for targeted treatment in the future, and it is conducive to improving the prognosis and survival rate.

In the last decade, we have witnessed a revolution in sequencing technology, which has enabled us to understand many concepts in genetics and genome biology [20]. Next-generation sequencing (NGS) technology solves the problem of detecting a large number of gene changes at one time. It can analyze point mutations, base deletion insertion mutations, gene copy number changes and gene fusion mutations at the same time and has the advantages of high accuracy, low sample demand and a short detection cycle. NGS has been demonstrated in different phase I and II trials, expanding our knowledge of the gastrointestinal microbiome. Many studies have shown that the diversity and community structure of gastrointestinal microbes change in malignant tumors such as GC [21], lung cancer [22] and intestinal tumors [23].

In this study, we collected gastric juice samples from 61 healthy people, 48 patients with early GC and 30 patients with advanced GC. We analyzed the community structure, alpha diversity, difference and correlation of gastric juice microbiota from healthy people and GC patients, early GC patients and advanced GC patients by bacterial 16 S rRNA detection. The research can provide data support for the screening of GC and the diagnosis and treatment of advanced GC by screening the bacterial flora of gastric juice associated with advanced GC.

## Materials and methods

### Data Acquisition

From December 2020 to August 2021, 48 patients with TNM stage I-II early GC and 30 patients with TNM stage III-IV advanced GC were admitted to the Department of Gastrointestinal Surgery and Oncology of Huzhou Central Hospital, and 61 healthy controls were recruited by the Health Examination Center.

The clinical trial and informed consent of the included patients were approved by the Ethics Committee of Huzhou Central Hospital (No. 20201205-02). The raw sequencing data have been deposited into the NCBI Sequence Read Archive (SRA) database under the accession number PRJNA890002 and PRJNA890143. The general condition of the patients was described in Additional file 1: Tables S1 and Additional file 2: Table S2.

Inclusion criteria for GC patients: (1) patients diagnosed with GC by pathology, and the clinical staging was according to the American Joint Cancer Committee (AJCC) cancer staging guidelines; (2) no surgery, chemotherapy, radiotherapy and other treatments; (3) patients or agent sign the informed consent.

Inclusion criteria for healthy controls: The healthy control group had no respiratory diseases, gastrointestinal diseases, oral diseases, malignant tumors or tumor-related symptoms in the past two years.

The exclusion criteria were as follows: (1) complications with other malignant tumors; (2) complications with serious cardiopulmonary diseases; (3) inability to undergo gastroscopy; (4) history of antibiotic, hormone or gastrointestinal microbiotic use 3 months before admission; and (5) chronic gastric ulcer, gastritis and other stomach diseases.

### Methods

#### Collection and processing of microbial specimens in gastric juice

Collection and processing of gastric juice samples: subjects were instructed to abstain from food and drink for more than 12 h before collection, and gastric contents were emptied. The next morning, 50 ml of sterilized water for injection was injected into the stomach through a gastroscope, 50 ml of gastric juice samples were extracted and labeled on the container for examination, and the bacterial flora of gastric juice was analyzed.

## MiSeq sequencing of the microbial genome

1. Genomic DNA extraction: Bacterial DNA was extracted from microbial samples of gastric juice using a bacterial DNA extraction kit. A NanoDrop2000 was used for DNA purity analysis. After selecting qualified samples, 16 S rRNA sequencing was commissioned by a professional company.
2. PCR amplification: Specific primers with barcodes were synthesized. The 16S rDNA primer sequences for the V1-V4 regions were 357F: 5ʹ-TACGGGAGGCAGCAG-3ʹ and 1114R: 5ʹ-GCAACGAGCGCAACCC-3ʹ. To ensure the accuracy and reliability of subsequent data analysis, two conditions should be met: (1) use low-cycle number amplification as far as possible; (2) ensure that the cycle number of amplification for each sample is consistent. A representative sample was randomly selected for the pre-experiment to ensure that the majority of samples could amplify the product at the right concentration at the lowest number of cycles. Polymerase chain reaction (PCR) products of the same sample were mixed and detected by 2% agarose gel electrophoresis. PCR products were recovered by gluing using an AxyPrepDNA gel recovery kit (AXYGEN company) and eluted with Tris_HCl. Electrophoresis was performed on 2% agarose. The PCR products were quantified with the QuantiFluor^™^-ST blue fluorescence Quantification system (Promega company) based on the preliminary results of electrophoresis and then mixed in proportion to the sequencing volume required for each sample.
3. MiSeq library construction: connecting “Y” joint; Magnetic bead screening was used to remove the self-connected segment of the joint; The library template was enriched by PCR amplification. Sodium hydroxide denatures, producing single-stranded DNA fragments.
4. MiSeq sequencing: one end of the DNA fragment is complementary to the primer base and fixed on the chip; the other end is randomly complementary to another primer nearby and is also fixed to form a “bridge”. PCR amplification was performed to generate DNA clusters. The DNA amplicon was linearized into a single strand. The modified DNA polymerase and four fluorescently labeled dNTPs were added to synthesize only one base per cycle. The surface of the reaction plate was scanned by laser to read the type of nucleotide that was aggregated in the first round of reaction of each template sequence. The “fluorophore” and the “termination group” were chemically cleaved to restore the viscosity of the 3’ end and continue to polymerize the second nucleotide. The fluorescence signal results collected in each round were counted to obtain the sequence of template DNA fragments.

 

## Bioinformatics analysis

OTU clustering analysis: The uparse (version 7.1) method was used for OTU clustering. The sequence similarity in OTUs was set to 97%, and the representative sequence of OTUs was obtained. uchime (version 4.2.40) was used to detect the chimeric sequences generated in PCR amplification and remove them from OTUs. The Usearch_global method was used to compare the map of optimized sequences back to the representative sequences of OTUs, and the sequence abundance tables of each OTU sample were obtained.

Diversity analysis: To study the microbial diversity of the fecal microbial community ecology of the sample, the diversity analysis of a single sample (Alpha diversity) can reflect the abundance and diversity of the microbial community, including a series of statistical analysis indices to estimate the species abundance and diversity of the environmental community. Mothur software (https://www.mothur.org/wiki/Download_mothur) was used to calculate the Chao abundance index and Ace index assessment flora. The Shannon index and Simpson index were calculated to evaluate the diversity of the flora.

Community structure analysis: The RDP classifier Bayesian algorithm was used to perform taxonomic analysis on 97% OTU representative sequences with similar levels, and the community composition of each sample was counted at each level (phylum, class, order, family, genus, species). Variance decomposition was used to reflect the differences of multiple groups of data on the two-dimensional coordinate map, and the two eigenvalues of the coordinate axes that could reflect the maximum variance value were used for PCA. The position of samples in each dimension was recorded, the contribution of each OTU to each principal component was calculated, and PCA statistical analysis and PCA diagram were performed using R language. PCoA first sorts a series of eigenvalues and eigenvectors, then selects the most important eigenvalues in the first few, displays them in the coordinate system, and uses R language for PCoA statistical analysis and mapping. Finally, Excel was used to draw a percentage stacked bar chart.

LEfSe multilevel discriminant analysis of species differences: LEfSe has a powerful identification function through biological significant differences. It then performs additional tests to assess whether these differences match the expected biological behavior. First, the nonparametric factorial Kruskal‒Wallis (KW) sum-rank test detects the characteristics of significant differences in abundance and finds the taxa that are significantly different from their abundance. Finally, LEfSe linear discriminant analysis (LDA) was used to estimate the magnitude of the effect of the abundance of each component (species) on the differential effect.

Build machine learning model: the differences in gastric flora for building elements, integrated application of intelligent colorectal cancer screening system was used to construct a prediction model for advanced GC by logistic regression (LR), random forest (RF), neural network (NN), support vector machine (SVM), CatBoost, and gradient boosted decision tree (GBDT).

## Statistical analysis

Statistical analysis was performed using SPSS V25.0 (SPSS Inc., Chicago, IL), For continuous variables, independent sample t test was used for single factor analysis between the two groups, and chi-square test was used for categorical variables. GraphPad Prism version 8.0 (San Diego, CA) and the Tutools platform (http://www.cloudtutu.com) were used for the preparation of graphs.

## Results

### Healthy people group and GC group

#### Descriptive analysis of Bacteria from healthy controls and GC patients

**Fig. 1:**
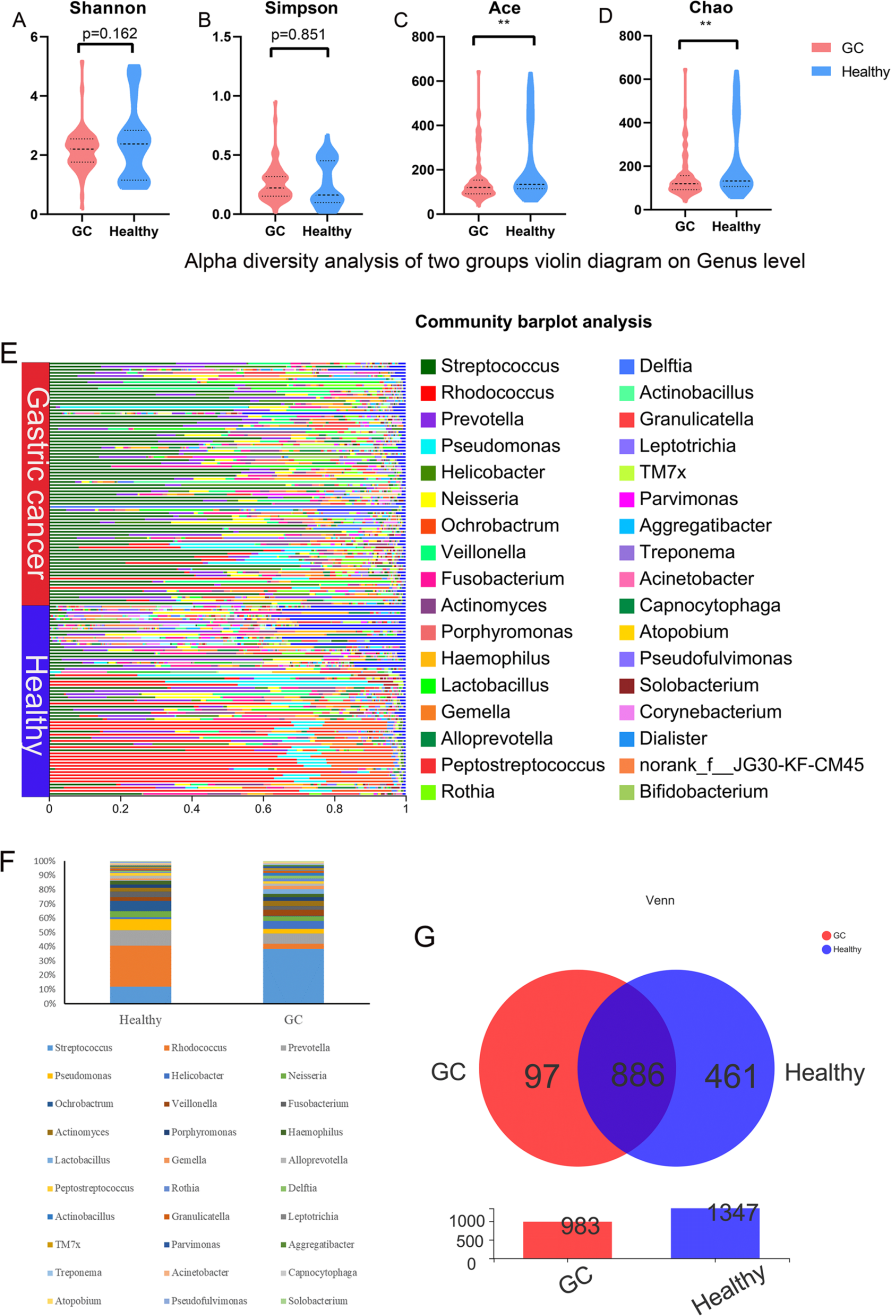
Alpha diversity analysis and composition of bacteria in gastric juice samples from healthy individuals and GC patients. **A** Shannon index. **B** Simpson index. **C** Ace index. **D** Chao index. * represents a significant difference between the two groups (p < 0.05). **E** Community composition of the bacterial community. The ordinate is the name of the sample, and the abscissa is the proportion of bacteria in the sample. Different colors of the column represent different species, and the length of the column represents the size of the proportion of the species. **F** A histogram of percentage accumulation drawn for the top 30 bacteria with the highest abundance in the two groups. **G** Venn diagram. Red is GC, blue is healthy, and the number of nonoverlapping species represents the number of species unique to the corresponding group

By comparing the bacterial diversity and community of the gastric juice of the healthy group and GC group, it was found that there was no difference in the diversity of bacteria between the two groups at the genus level (P > 0.05) (Fig. 1A–B). However, there were differences in the abundance of bacterial flora between the two groups (P < 0.05) (Fig. 1C–D). The sequencing depth was shown in Additional file 3: Table S3. The bacterial community structure of gastric juice was different between the two groups. *Streptococcus* was widely distributed in the GC group, and *Rhodococcus* was widely distributed in the healthy group (Fig. 1E**)**. The top five bacteria with the highest composition ratio in the two groups were *Streptococcus, Rhodococcus, Prevotella, Pseudomonas* and *Helicobacter*, and *Streptococcus* and *Helicobacter* accounted for more in the GC group. *Rhodococcus*, *Pseudomonas* and *Ochrobatrum* accounted for more in the healthy group (Fig. 1F). A Venn diagram showed that there were 886 common bacteria in the gastric juice of the two groups, 461 unique bacteria in the healthy group and 97 unique bacteria in the GC group (Fig. 1G).

## Differential bacteria between healthy controls and GC patients

**Fig. 2:**
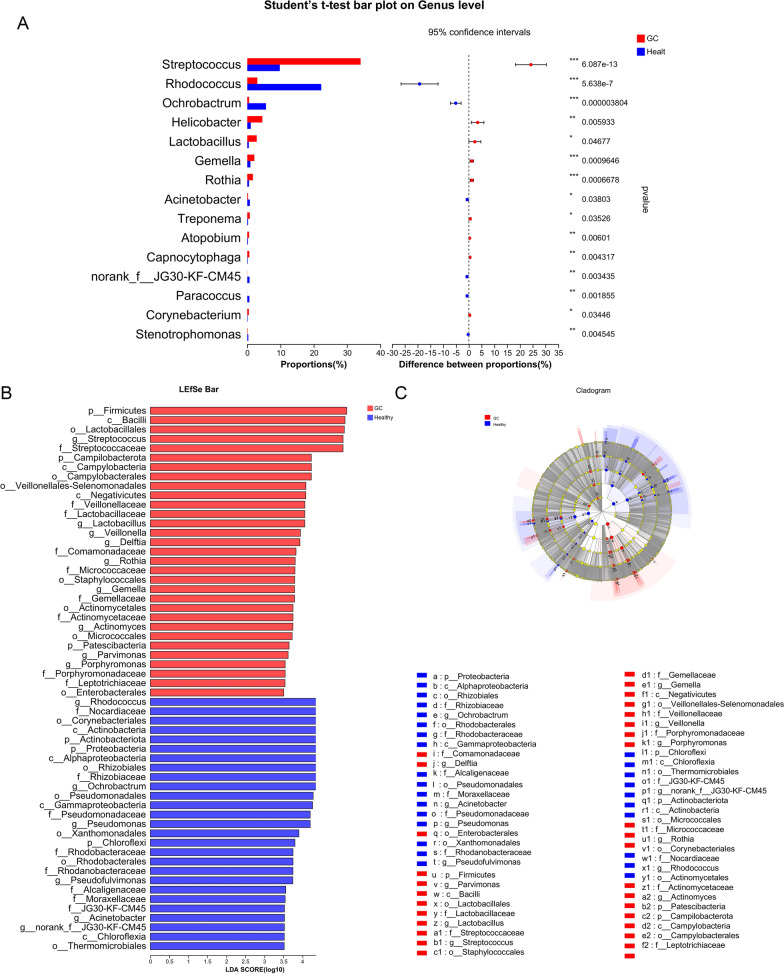
Multispecies difference test bar chart and diagram of different microflora between the two groups. **A** Student’s t test was used to test the hypothesis of species between the microbial communities of the two groups and evaluate the significance level of species abundance differences. P < 0.05 indicates a significant difference. The closer the line is to the middle, the smaller the standard deviation, and the better the central tendency. **B** The LDA score was obtained by linear regression analysis, and the greater the LDA score was, the greater the impact of bacterial abundance on the difference effect. An LDA score of more than 2 indicates a statistically significant difference (P < 0.05). **C** The graph shows LEfSe multistage species from the inner to the outer circle and represents the phylum, class, order, family, genus, and species of different unit levels. Different color nodes indicate the microbial groups that were significantly enriched in the corresponding groups and had a significant influence on the differences between groups. The pale-yellow nodes indicate the microbial groups that had no significant difference among different groups or had no significant effect on the difference between groups

The differences in bacteria in the gastric juice of the two groups were analyzed, and 15 bacteria, including *Streptococcus, Rhodococcus and Ochrobactrum*, were screened out (Fig. 2A). LEfSe analysis was used to compare the different bacteria between the two groups. The results showed that the characteristic bacteria of the healthy group were 112 species, such as *Rhodococcus, Ochrobactrum* and *Pseudomonas*, indicating that these bacteria were more important in the healthy group but less important in the GC group. The characteristic bacteria of the GC group were 40 species, such as *Streptococcus, Veillonella* and *Lactobacillus* (Fig. 2B–C).

### **Correlation of differentially abundant bacteria between healthy people and GC patients**

**Fig. 3:**
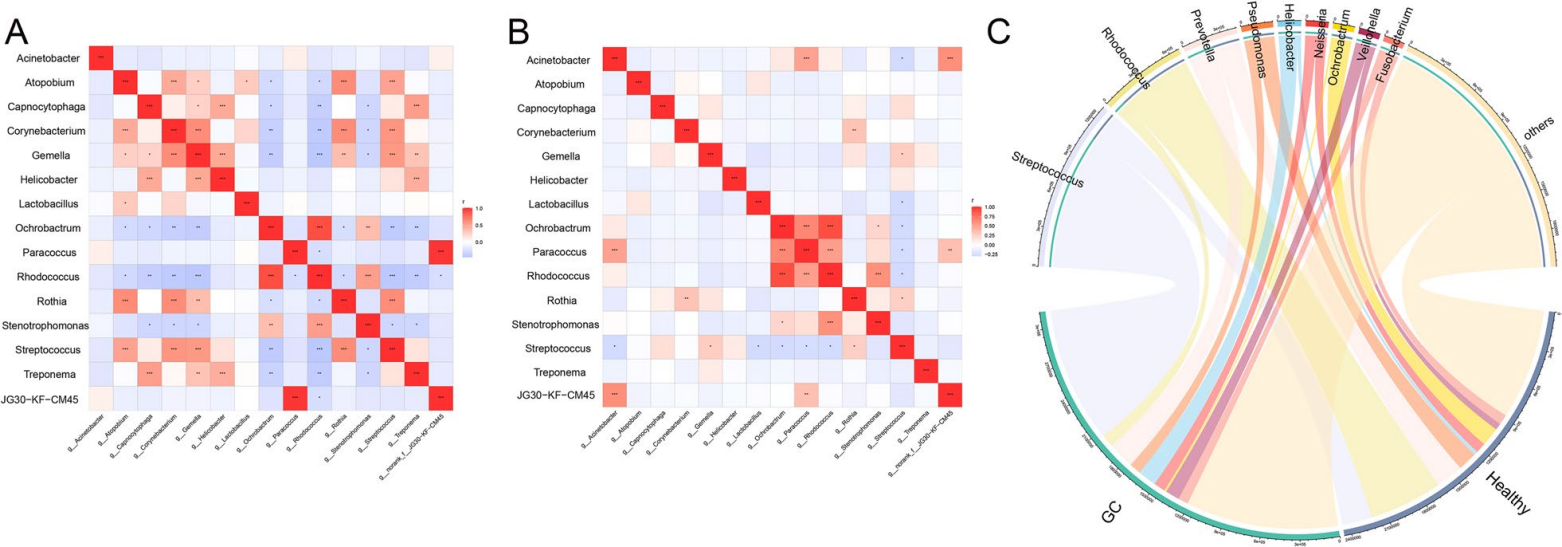
Correlation analysis of different bacteria within and between groups. The numerical matrix of the two groups of different bacteria is visually displayed through the heatmap. The color change reflects the data information, and the color depth represents the correlation. The redder the color is, the higher the correlation between the two bacteria. **A** Intragroup bacterial correlation heatmap of the healthy group. The Pearson coefficient was used to calculate the correlation between the bacteria. The shade of color indicates the size of the data value. Pearson correlation coefficients are indicated in the figure. *0.01 < p < 0.05; **0.001 < p ≤ 0.01; ***p ≤ 0.001. **B** Intragroup bacterial correlation heatmap of the GC group. **C** Chord diagram. One side of the circle is the species name, and the other side is the sample name, which is represented by different colors. The species abundance is displayed as a percentage

To further clarify the relationship between different bacteria in GC, the correlation between different bacteria was analyzed. The significantly correlated differences in the healthy group were *Ochrobactrum* and *Rhodococcus* (r = 0.916, P < 0.001) *Paracoccus* and *JG30 − KF − CM45* (r = 0.987, P < 0.001) (Fig. 3A); The significantly associated differentially abundant bacteria in the GC group were *Ochrobactrum* and *Paracoccus *(r = 0.650, P < 0.001), *Ochrobactrum* and *Rhodococcus* (r = 0.901, P < 0.001), *Paracoccus* and *Rhodococcus* (r = 0.537, P < 0.001) (Fig. 3B). Bacteria such as *Streptococcus* and *Helicobacter* were more closely related to GC, and bacteria such as *Rhodococcus* and *Pseudomonas* were more closely related to healthy people (Fig. 3C).

### Construction of a prediction model for GC

**Fig. 4:**
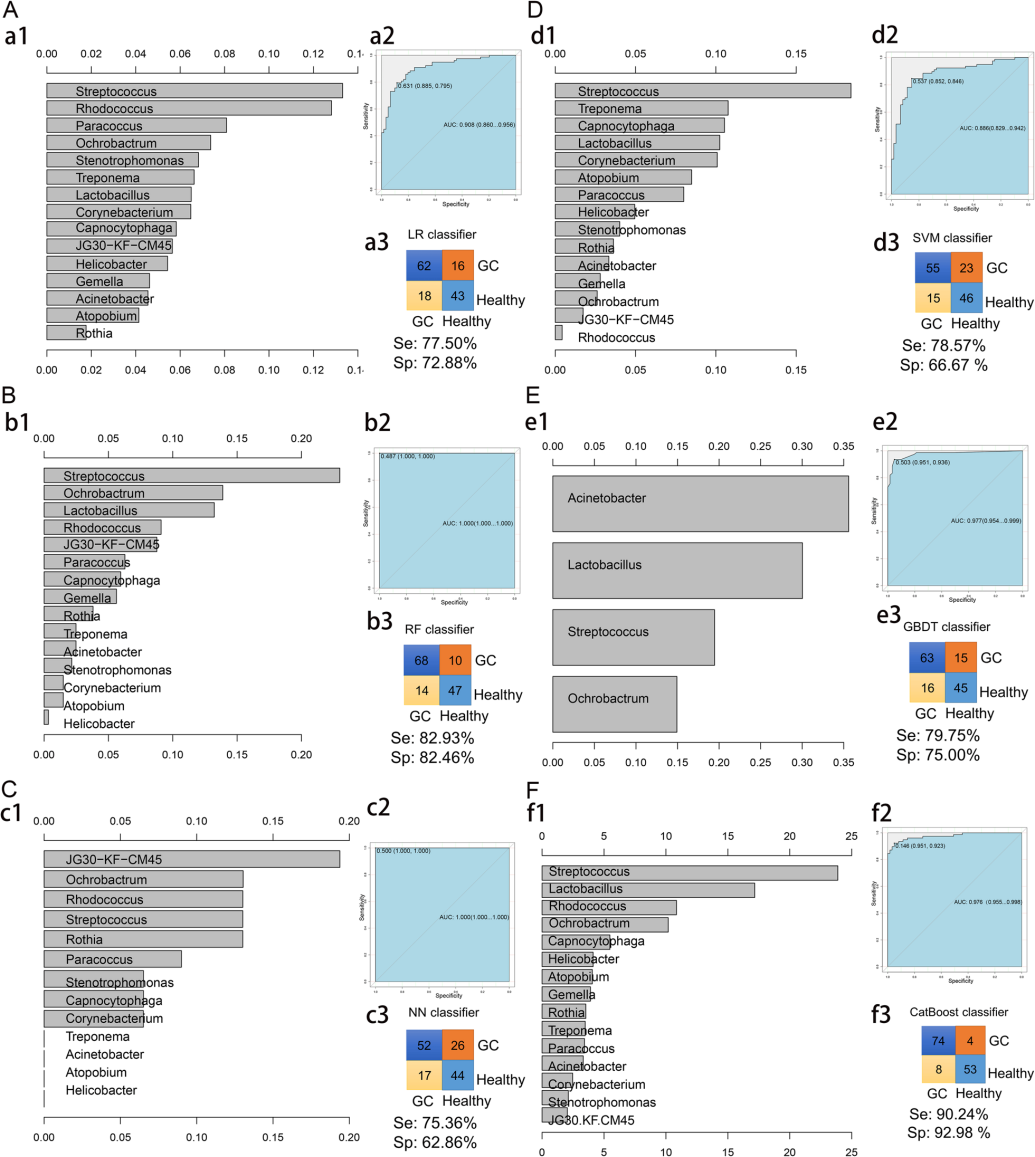
Graph of the GC risk prediction model. Six models were constructed using the top 30 most abundant bacteria. We used the relevant functions in the rminer Package (version 1.4.5) of R language for modeling analysis and used the fit function for modeling the variable importance calculation. **A–F** are the LR model (**A**), RF model (**B**), NN model (**C**), SVM model (**D**), GBDT model (**E**) and CatBoost model (**F**). The left panel (a1, b1, c1, d1, e1, f1) show the variable importance histogram of the model, the upper right panel (a2, b2, c2, d2, e2, f2) show the AUC curve of the model, and the lower right panel (a3, b3, c3, d3, e3, f3) show the accuracy of the model

Six machine learning algorithms were used to construct a logistic regression model, random forest model, neural network model, support vector machine model, gradient lifting tree model, and CatBoost model. Figure 4A Logistic regression model. The characteristic bacteria screened based on the logistic regression algorithm were *Streptococcus, Paracoccus, Rhodococcus*, etc., AUC = 0.908 and prediction accuracy of 75.54%; Fig. 4B Random forest model. The characteristic bacteria screened based on the random forest algorithm were *Streptococcus, Lactobacillus, Ochrobactrum*, etc., AUC = 1.000 and prediction accuracy of 82.73%; Fig. 4C Neural network model, the characteristic bacteria screened based on the neural network algorithm were *g__norank_f__JG30 − KF − CM45, Ochrobactrum, Rhodococcus*, AUC = 1.000, and the prediction accuracy was 69.06%. Figure 4D Support vector machine model. The characteristic bacteria screened based on the support vector machine algorithm were *Streptococcus, Treponema* and *Capnocytophaga*, AUC = 0.886 and prediction accuracy of 72.66%. Figure 4E Gradient lifting tree model. The characteristic bacteria selected based on the gradient lifting tree algorithm were *Acinetobacter, Lactobacillus* and *Streptococcus*, AUC = 0.977 and prediction accuracy of 77.70%. Figure 4F CatBoost model. The characteristic bacteria screened based on the CatBoost algorithm were *Streptococcus, Lactobacillus* and *Rhodococcus*, AUC = 0.976. Therefore, the random forest model is the best model for predicting GC.

## Healthy group, early GC and advanced GC group

### Descriptive analysis of bacteria from the healthy group, early GC and advanced GC

**Fig. 5:**
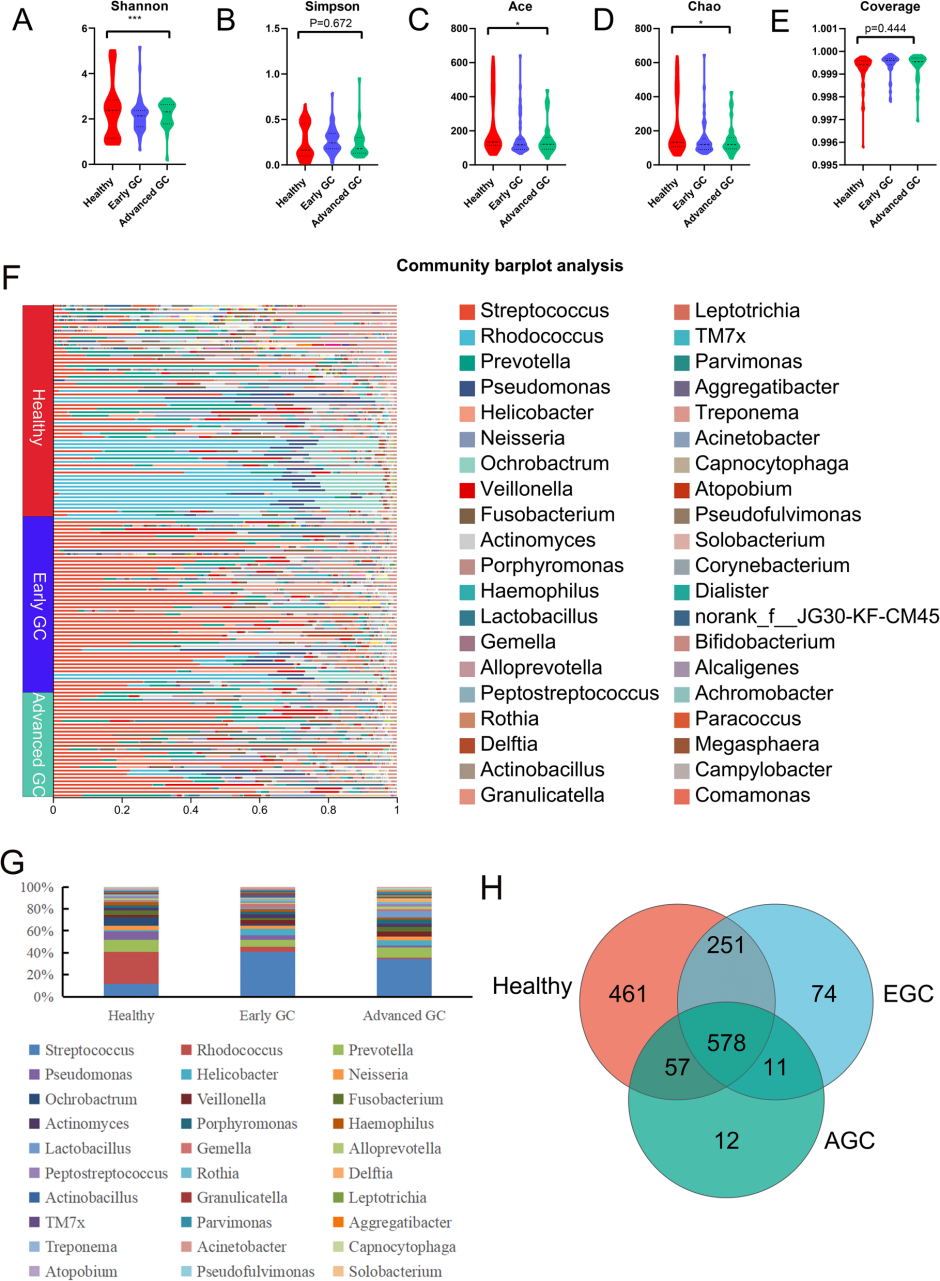
Alpha diversity analysis and composition of bacteria in gastric juice samples from healthy group, early GC and advanced GC group. The alpha diversity of bacteria among the three groups of gastric juice samples was analyzed at the genus level, and a violin diagram was constructed. Red represents the healthy group, Blue represents the early GC group, and green represents the advanced GC group. **A** Shannon index. **B** Simpson index. **C** Ace index. **D** Chao index. **E** Coverage index. * represents a significant difference in the three groups (p < 0.05). **F** Community composition of the gastric juice bacterial community. The ordinate is the name of the sample, and the abscissa is the proportion of bacteria in the sample. Different colors of the column represent different species, and the length of the column represents the size of the proportion of the species. **G** A histogram of percentage accumulation drawn for the top 30 bacteria with the highest abundance in the three groups. **H** Venn diagram. Healthy group is shown in red, early gastric cancer is shown in blue, and advanced gastric cancer is shown in green, and the number of nonoverlapping species represents the number of species unique to the corresponding group

By comparing the bacterial diversity and community fabric of gastric juice of healthy group, early GC and advanced GC, difference in bacterial diversity and bacterial flora abundance was found among the three groups at the genus level (P < 0.05) (Fig. 5A–E). The sequencing depth was shown in Additional file 4: Table S4. The bacterial community structures in the gastric juice of the three groups were different, and *Streptococcus* was widely distributed in the GC groups (Fig. 5F). The top five bacteria with the highest composition ratio in the three groups were *Streptococcus, Rhodococcus, Prevotella, Pseudomonas* and *Helicobacter, Streptococcus* and *Rhodococcus* were significantly different among the three groups (Fig. 5G). The number of bacteria common to the three groups was 578, the number of bacteria unique to the healthy group was 461, the number of bacteria unique to the early GC group was 74, and the number of bacteria unique to the advanced GC group was 12. (Fig. 5H).

### Differential bacteria in the healthy group, early GC and advanced GC

**Fig. 6:**
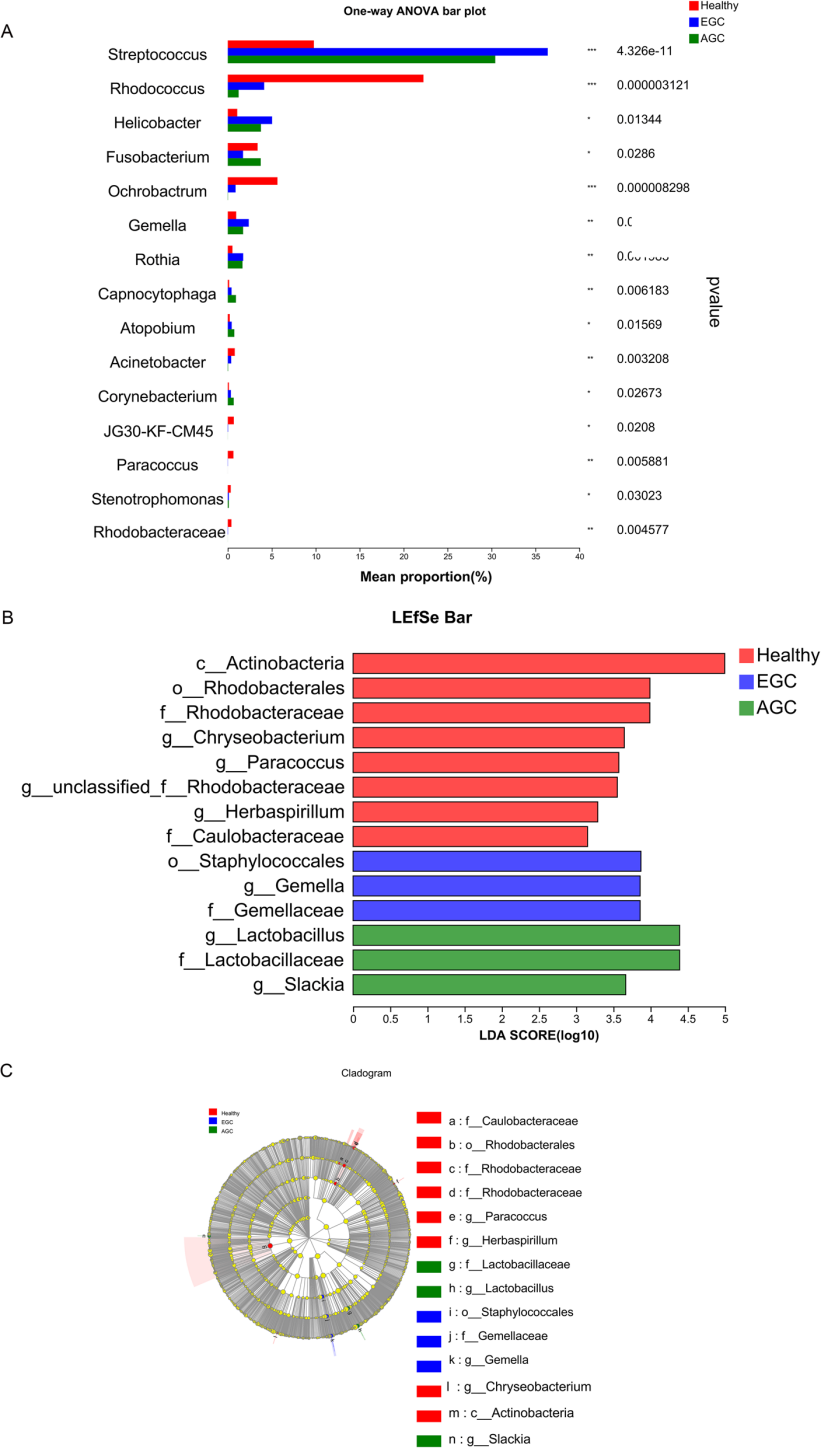
Multispecies difference test bar chart and diagram of different microflora in the three groups. **A** The statistical method of Student’s t test was used to test the hypothesis of the species between the microbial communities of the three groups of samples and evaluate the significance level of the difference in species abundance. P < 0.05 indicates a significant difference. The closer the line is to the middle, the smaller the standard deviation and the better the central tendency. **B** The LDA score was obtained by linear regression analysis, and the greater the LDA score was, the greater the impact of bacterial abundance on the difference effect. An LDA score of more than 2 indicates a statistically significant difference (P < 0.05). **C** The graph shows LEfSe multistage species from the inner to the outer circle and represents the phylum, class, order, family, genus, and species of different unit levels. Different color nodes indicate the microbial groups that were significantly enriched in the corresponding groups and had a significant influence on the differences. The pale-yellow nodes indicate the microbial groups that had no significant difference among different groups

The differences in bacteria in gastric juice among the three groups were analyzed. 15 differential bacteria such as *Streptococcus, Rhodococcus, Ochrobactrum, Fusobacterium, and Helicobacter* were screened out (Fig. 6 A). LEfSe analysis was used to compare the different bacteria among the three groups, and the results showed that the characteristic bacteria of the early GC group were *Staphylococcales, Gemella, Gemellaceae*. These results indicated that these bacteria were more important in the early GC group. The characteristic bacteria of the advanced GC group were *Lactobacillus, Lactobacillaceae, Slackia.* (Fig. 6B C).

### Correlation of differentially abundant bacteria in healthy group, early GC and advanced GC

**Fig. 7:**
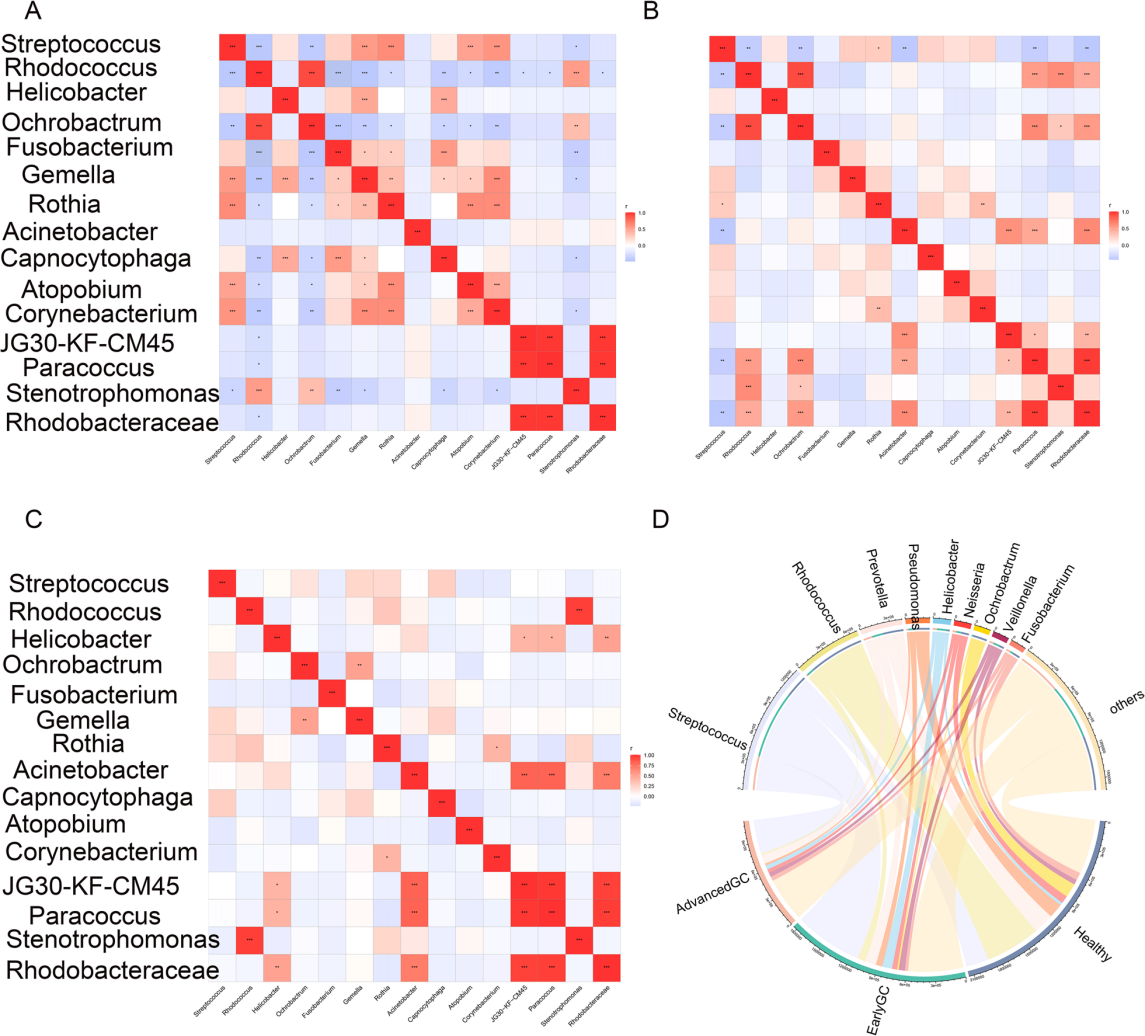
Correlation analysis of different bacteria within and among three groups. **A** Intragroup bacterial correlation heatmap of the healthy group. The Pearson coefficient was used to calculate the correlation between the bacteria. The shade of color indicates the size of the data value. Pearson correlation coefficients are indicated in the figure. *0.01 < p < 0.05; **0.001 < p ≤ 0.01; ***p ≤ 0.001. **B** Intragroup bacterial correlation heatmap of the early GC group. **C** Intragroup bacterial correlation heatmap of the advanced GC group. **D** Chord diagram. One side of the circle is the species name, and the other side is the sample name, which is represented by different colors. The species abundance is displayed as a percentage

To further clarify the relationship between different bacteria in advanced GC, the correlation between different bacteria was analyzed. The significantly associated differentially abundant bacteria in the healthy group were *Ochrobactrum* and *Rhodococcus* (r = 0.916, P < 0.001) (Fig. 7A); The significantly associated differentially abundant bacteria in the early GC group were *Ochrobactrum* and *Rhodococcus* (r = 0.926, P < 0.001) (Fig. 7B); The significantly associated differentially abundant bacteria in the advanced GC group were *Stenotrophomonas* and *Rhodococcus* (r = 0.626, P < 0.001) (Fig. 7C). *Prevotella* was more correlated with healthy group, *Helicobacter* was more correlated with early GC, and *Streptococcus* was more correlated with advanced GC (Fig. 7D).

## Discussion

The stomach plays an important role in maintaining gastrointestinal health by providing a barrier against gastrointestinal infectious disease pathogens. Patients with GC of the stomach contain a large number of microbes, and microorganisms play an important role in the occurrence and development of GC. Screening specific gastric microbiota provides a new direction for further exploring the early screening and diagnosis of GC and the treatment of advanced GC. In this study, gastric juice samples were collected from 61 healthy people, 48 patients with early GC and 30 patients with advanced GC.

Our study found that there was difference in microbiota diversity and community richness in the healthy group, early GC and advanced GC group. Dai et al. [24] found that the diversity and richness of microbes in GC was higher than that in non-tumor tissue. Ferreira et al. [25] performed a retrospective study of the gastric microflora of 54 patients with GC and 81 patients with chronic gastritis using 16 S rRNA gene profiling and found that GC flora was characterized by decreased microbial diversity, decreased *HP* abundance, and enrichment of other bacteria genera. The main genera found in the stomach were *Prevotella, Streptococcus, Vermicelli, Roxella* and *Haemophilus* [26]. In this study, *Streptococcus, Rhodococcus, Precotella, Pseudomonas* and *Helicobacter* were widely distributed in GC patients, with high abundance in some samples. Abate et al. [27] found that *Helicobacter, Lactobacillus, Streptococcus, Prevotella* and *Bacteroides* were significantly more enriched in GC samples. As the disease progresses to more severe stages, the advantages of *HP* begin to be replaced by other bacteria, including *Streptococcus, Prevotella, Achromobacter, Citrobacter, Clostridium, Rhodococcus, lactic acid bacteria* and *Phyllobacter* [28]. In the study, the abundance of *Helicobacter* and *Streptococcus* were highest in early GC and lowest in healthy group. But Dai D, et al. [24] found that the abundance of *HP* increased in non-tumor tissues, while the abundance of *Streptococcus, Bacteroides, Prevotella* increased in tumor tissues. Dicksved et al. [29] conducted gene sequence analysis on the adjacent gastric mucosa tissues of 10 patients with GC and the gastric mucosa tissues of 5 patients with dyspepsia and found that the number of *HP* in the gastric mucosa of GC patients was lower, and the main bacteria were *Streptococcus* and *Prevotella*. Liu et al. [30] found that *Prevotella melaninogenica* and *Streptococcus anginosus* were increased in the tumoral microhabitat, and *HP, Prevotella copri* and *Bacteroides uniformis* were significantly decreased. It was suggested that the differences in metabolome profiles between GC tumors and matched non-tumor tissues may be partly due to the collective activities of *HP, Lactobacillus* and other bacteria, which ultimately affect the occurrence and progression of GC.

Hu et al. [31] showed that compared with superficial gastritis, the microbiome of GC was characterized by the enrichment of a variety of bacterial genera and species including genera *Neisseria, Alloprevotella*, and *Aggregatibacter*, species *Streptococcus_mitis_oralis_pneumoniae*. The study found that *Neisseria, Alloprevotella* and *Aggregatibacter* accounted for the highest proportion in advanced GC patients. The results showed that *Streptococcus* was the bacterium with the highest proportion in early GC and advanced GC, and this was consistent with the findings of Zhou et al. [32]. But Aviles-Jimenez [14] found that the amount of *Streptococci* in the GC patients decreased, and the diversity of bacteria gradually decreased during the transition from nonatrophic gastritis to intestinal metaplasia and intestinal GC. In the present study, the abundance of *Lactobacillus* was increased in the advanced GC group and *Fusobacterium* was abundant in advanced GC and healthy group But *Fusobacterium nucleatum* has been found in GC [33, 34], and the presence of *Fusobacterium nucleatum* in tumors was thought to be associated with poor survival [35]. In conclusion, Gastric microbiome plays an important role in the gastric carcinogenesis. The community structure and diversity of gastric microbiota in patients with advanced GC have changed, and the gastric microbiota can be used to distinguish early and advanced GC patients.

Bacteria can promote anti-tumor immune responses through a variety of mechanisms, such as triggering T cell responses to bacterial antigens, or inducing tumor-specific antigen recognition through small metabolites mediating systemic effects on the host [36]. *HP* infection induced nonspecific inhibition of circulating T cells [37]. This group of bacteria, particularly *lactic acid bacteria, Streptococcus* increased the number of CD3 + T, CD4 + T, and natural killer cells [38]. The study found that *Lactobacillus* differed among the three groups. Lactic acid can promote chemotherapy resistance [39] and promote tumor growth [40]. Lactic acid bacteria such as S*treptococcus, Lactobacillus* and *Lactococcus* have been linked to the progression of GC. They can influence the development of GC in a variety of ways, including increasing N-nitroso compounds causing DNA damage and regulating the expression of key molecules important in cancer development [41]. They found that patients with GC exhibit a genotoxic potential for abiotic microbial communities. Microbial communities found in GC showed increased nitrosation function, consistent with increased genotoxic potential [25]. The study found *Veillonella* was relatively higher in the GC group. Studies have found that the presence of *Veillonella* was related to the tumor response to nivolumab [5]. A unique group of bacteria, including *Streptococcus, Parvoomonas, Prevotella, Roche* and *Bacillus*, were potential therapeutic targets for the prevention of GC [42]. Recent consensus and meta-analysis reports indicate that eradication of *HP* reduces the incidence of GC by a factor of 0.55 [43]. It follows that changes in the gastric microbiome may need to be considered to improve the therapeutic effect.

Some scholars have found that *Streptococcus anginosus* and *Streptococcus constellatus* are enriched in the feces of precancerous and early GC patients, and therefore they can be used as a means for early screening of GC. However, the collection method of fecal microorganisms does not take into account the influence of the upper digestive tract. In this study, gastric juice microbiota was studied in healthy people and GC patients (early and advanced GC) to provide a reference for the method of obtaining gastric juice samples. Because the difficulty of collecting specimens of gastric juice is easily affected by many factors, such as the intubation pathway, the depth of the tube, client postures, and the absorbing method, the study of gastric juice sample collection and processing of the standardization process has been established, and a standard gut microbe noninvasive test method that is simple and convenient to use to determine the potential of GC occurrence mechanism provides a new direction. This study explored new methods of human digestive tract bacteromics from the perspective of microorganisms, screened harmful bacteria associated with GC progression, and provided data support for the study of the mechanism of GC progression.

The gut microbiota is an exploratory biomarker for GC immunotherapy, searching for the microbiota that can inhibit the progression of GC and providing a research direction for the microbial treatment of advanced GC. Analyzing the microbial community composition and structure in the gastric juice of healthy people, early GC patients and patients with advanced GC can provide data support for the screening of early GC and the diagnosis and treatment of advanced GC.

There are some limitations in this study. The sample size in this study was insufficient, and there may be false negative results. However, this study provides ideas and references for subsequent multicenter and large-scale studies to elucidate the relationship between the occurrence and development of GC and gastric juice microbes. Although potential procancer bacteria and anticancer bacteria have been screened for GC, the specific mechanism of harmful bacteria, such as *Streptococcus*, leading to advanced GC is unclear and requires further molecular experiments for verification. Further research is needed to determine whether probiotics such as *Lactobacillus* can be used as adjuvant drugs in the treatment of GC.

## Supplementary Information

Additional file 1: Table S1. Clinical information on patients with healthy and GC.

Additional file 2: Table S2. Clinical information on patients with Healthy group, Early GC and Advanced GC.

Additional file 3: Table S3. Table of diversity indices about healthy and GC. The indexes of community richness were chao and ace. The indexes of community diversity were shannon, simpson and coverage.

Additional file 4: Table S4. Table of diversity indices about Healthy group, Early GC and Advanced GC. The indexes of community richness were chao and ace. The indexes of community diversity were shannon, simpson and coverage.

## Data Availability

The datasets generated for this study can be accessed from the NCBI Sequence Read Archive (SRA) database under the accession number PRJNA890002 and PRJNA890143 (http://www.ncbi.nlm.nih.gov/bioproject/890002, http://www.ncbi.nlm.nih.gov/bioproject/890143). The data will be released to the public in October 2026.

## Abbreviations

GC: Gastric cancer
IARC: International agency for research on cancer
WHO: World Health Organization
HP: *Helicobacter pylori*
NGS: Next-generation sequencing
PCoA: Principal coordinate analysis
PCR: Polymerase chain reaction
LDA: Linear discriminant analysis
LR: Logistic regression
RF: Random forest
NN: Neural network
GBDT: Gradient boosted decision tree
SVM: Support vector machine

## References

[1] Balea AM, Cruce R, chenker R A (2019). Correlations between clinicopathological features and the vegetative nervous system in gastric Cancer [J]. Curr Health Sci J.

[2] Yuka O, Sachio F, Takahisa Y (2020). Peripheral blood platelet-lymphocyte ratio is good predictor of chemosensitivity and prognosis in gastric cancer patients [J]. Cancer Manag Res.

[3] Sung H, Ferlay J, Siegel RL (2021). Global cancer statistics 2020: GLOBOCAN estimates of incidence and mortality worldwide for 36 cancers in 185 countries. CA Cancer J Clin.

[4] Yamamoto H, Watanabe Y, Oikawa R (2016). BARHL2 methylation using gastric wash DNA or gastric juice exosomal DNA is a useful marker for early detection of gastric cancer in an H. pylori-independent Manner [J]. Clin Transl Gastroenterol.

[5] Li K, Zhang A, Li X (2021). Advances in clinical immunotherapy for gastric cancer [J]. Biochim Biophys Acta Rev Cancer.

[6] Durães C, Almeida GM, Seruca R (2014). Biomarkers for gastric cancer: prognostic, predictive or targets of therapy [J]. Virchows Archiv An Int J Pathol.

[7] Liu HS, Xiao HS (2014). MicroRNAs as potential biomarkers for gastric cancer [J]. World J Gastroenterol Wjg.

[8] Jiang F, Zhou HY, Zhou LF (2019). MicroRNA-421 promotes inflammatory response of fibroblast-like synoviocytes in rheumatoid arthritis by targeting SPRY1 [J]. Eur Rev Med Pharmacol Sci.

[9] Tsuda A, Suda W, Morita H (2015). Influence of proton-pump inhibitors on the luminal microbiota in the gastrointestinal tract [J]. Clin Transl Gastroenterol.

[10] Nardone G, Compare D, Rocco A (2017). A microbiota-centric view of diseases of the upper gastrointestinal tract [J]. Lancet Gastroenterol Hepatol.

[11] Sha S, Ni L, Stefil M (2020). The human gastrointestinal microbiota and prostate cancer development and treatment [J]. Investig Clin Urol.

[12] Lertpiriyapong K, Whary MT, Muthupalani S (2014). Gastric colonisation with a restricted commensal microbiota replicates the promotion of neoplastic lesions by diverse intestinal microbiota in the Helicobacter pylori INS-GAS mouse model of gastric carcinogenesis [J]. Gut.

[13] Eun CS, Kim BK, Han DS (2014). Differences in gastric mucosal microbiota profiling in patients with chronic gastritis, intestinal metaplasia, and gastric cancer using pyrosequencing methods [J]. Helicobacter.

[14] Aviles-Jimenez F, Vazquez-Jimenez F, Medrano-Guzman R (2014). Stomach microbiota composition varies between patients with non-atrophic gastritis and patients with intestinal type of gastric cancer [J]. Sci Rep.

[15] Yu G, Torres J, Hu N (2017). Molecular characterization of the human stomach microbiota in gastric Cancer Patients[J]. Front Cell Infect Microbiol.

[16] Sung J, Kim N, Kim J (2016). Comparison of gastric microbiota between gastric juice and mucosa by next generation sequencing Method [J]. J Cancer Prev.

[17] Kuo CH, Wang SS, Lu CY (2013). Long-term use of probiotic-containing yogurts is a safe way to prevent helicobacter pylori: based on a mongolian gerbil’s model [J]. Biochem Res Int.

[18] Paroni Sterbini F, Palladini A, Masucci L (2016). Effects of proton pump inhibitors on the gastric mucosa-associated microbiota in dyspeptic patients [J]. Appl Environ Microbiol.

[19] von Rosenvinge EC, Song Y, White JR (2013). Immune status, antibiotic medication and pH are associated with changes in the stomach fluid microbiota [J]. ISME J.

[20] Meldrum C, Doyle MA, Tothill RW (2011). Next-generation sequencing for cancer diagnostics: a practical perspective[J]. Clin Bio-chem Rev.

[21] Nardone G, Compare D (2015). The human gastric microbiota: is it time to rethink the pathogenesis of stomach diseases? [J]. United Eur Gastroenterol J.

[22] Gui QF, Lu HF, Zhang CX (2015). Well-balanced commensal microbiota contributes to anti-cancer response in a lung cancermouse model [J]. Genet Mol Res.

[23] Manzat-Saplacan RM, Mircea PA, Balacescu L (2015). Can we change our microbiome to prevent colorectal cancer development? [J]. Acta Oncol.

[24] Dai D, Yang Y, Yu J, Dang T, Qin W, Teng L, Ye J, Jiang H (2021). Interactions between gastric microbiota and metabolites in gastric cancer [J]. Cell Death Dis.

[25] Ferreira RM, Pereira-Marques J, Pinto-Ribeiro I, Costa JL, Carneiro F, Machado JC, Figueiredo C (2018). Gastric microbial community profiling reveals a dysbiotic cancer-associated microbiota [J]. Gut.

[26] Li TH, Qin Y, Sham PC, Lau KS, Chu KM, Leung WK (2017). Alterations in gastric microbiota after H. pylori eradication and in different histological stages of gastric carcinogenesis [J]. Sci Rep.

[27] Abate M, Vos E, Gonen M, Janjigian YY, Schattner M, Laszkowska M, Tang L, Maron SB, Coit DG, Vardhana S, Vanderbilt C, Strong VE (2022). A novel microbiome signature in gastric cancer: a two independent cohort retrospective analysis [J]. Ann Surg..

[28] Waskito LA, Rezkitha YAA, Vilaichone RK, Sugihartono T, Mustika S, Dewa Nyoman Wibawa I, Yamaoka Y, Miftahussurur M (2022). The role of non-helicobacter pylori bacteria in the pathogenesis of gastroduodenal diseases [J]. Gut Pathog.

[29] Camargo MC, Figueiredo C, Machado JC (2019). Review: gastric malignancies: basic aspects [J]. Helicobacter.

[30] Liu X, Shao L, Liu X (2019). Alterations of gastric mucosal microbiota across different stomach microhabitats in a cohort of 276 patients with gastric cancer [J]. EBioMedicine.

[31] Hu YL, Pang W, Huang Y (2018). The gastric microbiome is perturbed in advanced gastric adenocarcinoma identified through shotgun metagenomics [J]. Front Cell Infect Microbiol.

[32] Zhou CB, Pan SY, Jin P (2022). Fecal signatures of streptococcus anginosus and streptococcus constellatus for noninvasive screening and early warning of gastric cancer [J]. Gastroenterology.

[33] Yu J, Feng Q, Wong SH, Zhang D, Liang QY, Qin Y (2017). Metagenomic analysis of faecal microbiome as a tool towards targeted non-invasive biomarkers for colorectal cancer [J]. Gut.

[34] Hsieh YY, Tung SY, Pan HY, Yen CW, Xu HW, Lin YJ (2018). Increased abundance of clostridium and fusobacterium in gastric microbiota of patients with gastric cancer in Taiwan [J]. Sci Rep.

[35] de Carvalho AC, de Mattos PL, Datorre JG, dos Santos W, Berardinelli GN, Matsushita MM (2019). Microbiota profile and impact of fusobacterium nucleatum in colorectal cancer patients of barretos cancer hospital [J]. Front Oncol.

[36] Zitvogel L, Ma Y, Raoult D, Kroemer G, Gajewski TF (2018). The microbiome in cancer immunotherapy: diagnostic tools and therapeutic strategies [J]. Science.

[37] Nasr R, Shamseddine A, Mukherji D, Nassar F, Temraz S (2020). The crosstalk between microbiome and immune response in GC [J]. Int J Mol Sci.

[38] Qi YF, Sun JN, Ren LF, Cao XL, Dong JH, Tao K, Guan XM, Cui YN, Su W (2019). Intestinal microbiota is altered in patients with gastric cancer from Shanxi Province, China [J]. Dig Dis Sci.

[39] Wagner W, Ciszewski WM, Kania KD (2015). L- and D-lactate enhance DNA repair and modulate the resistance of cervical carcinoma cells to anticancer drugs via histone deacetylase inhibition and hydroxycarboxylic acid receptor 1 activation [J]. Cell Commun Signal.

[40] Voss DM, Spina R, Carter DL, Lim KS, Jeffery CJ, Bar EE (2017). Disruption of the monocarboxylate transporter-4-basigin interaction inhibits the hypoxic response, proliferation, and tumor progression [J]. Sci. Rep.

[41] San-Millán I, Brooks GA (2017). Reexamining cancer metabolism: lactate production for carcinogenesis could be the purpose and explanation of the Warburg effect [J]. Carcinogenesis.

[42] Sung JJY, Coker OO, Chu E, Szeto CH, Luk STY, Lau HCH, Yu J (2020). Gastric microbes associated with gastric inflammation, atrophy and intestinal metaplasia 1 year after helicobacter pylori eradication [J]. Gut.

[43] Liou JM, Malfertheiner P, Lee YC, Sheu BS, Sugano K, Cheng HC, Yeoh KG, Hsu PI, Goh KL, Mahachai V, Gotoda T, Chang WL, Chen MJ, Chiang TH, Chen CC, Wu CY, Leow AH-R, Wu JY, Wu DC, Hong TC, Lu H, Yamaoka Y, Megraud F, Chan FKL, Sung JJ, Lin JT, Graham DY, Wu MS, El‑Omar EM (2020). Asian pacific alliance on helicobacter and Microbiota (APAHAM) Screening and eradication of helicobacter pylori for gastric cancer prevention: the Taipei global consensus [J]. Gut.

