# Supplementary material for: Analysis of bacterial diversity and community structure in gastric juice of patients with advanced gastric cancer
Source: Discov Oncol. 2023 Jan 20;14:7. doi: 10.1007/s12672-023-00612-7 (PMC9860007; doi:10.1007/s12672-023-00612-7)
Supplement: Supplementary file 1 — Additional file 1: Table S1. Clinical information on patients with healthy and GC. [file 12672_2023_612_MOESM1_ESM.docx]

|  | Healthy | GC | P |
| --- | --- | --- | --- |
| N | 61 | 78 | — |
| Age | 51.61±11.68 | 67.97±9.24 | 0.000 |
| BMI (kg/m2) | 23.96±3.3 | 22.24±3.32 | 0.004 |
| CA125 | 11.66±5.79 | 19.49±30.93 | 0.153 |
| CA153 | 10.73±4.5 | 8.75±5.12 | 0.127 |
| CA724 | 3.13±2.87 | 3.5±4.66 | 0.695 |
| CA199 | 10.36±15.15 | 340.48±1877.95 | 0.158 |
| CEA | 1.98±1.21 | 7.56±21.16 | 0.025 |
| AFP | 2.78±1.06 | 68.61±573.43 | 0.493 |
| Male | 37 | 58 | 0.085 |
| Female | 24 | 20 |  |
| Smoking history | 16 | 22 | 0.795 |
| Drinking history | 17 | 33 | 0.078 |
| Hypertension history | 14 | 31 | 0.031 |
| Diabetes history | 5 | 4 | 0.478 |

**Additional file 1 Table 1 Clinical information on patients with healthy and GC**
