# Supplementary material for: Analysis of bacterial diversity and community structure in gastric juice of patients with advanced gastric cancer
Source: Discov Oncol. 2023 Jan 20;14:7. doi: 10.1007/s12672-023-00612-7 (PMC9860007; doi:10.1007/s12672-023-00612-7)
Supplement: Supplementary file 2 — Additional file 2: Table S2. Clinical information on patients with Healthy group, Early GC and Advanced GC. [file 12672_2023_612_MOESM2_ESM.docx]

**Additional file 2 Table 2 Clinical information on patients with Healthy group, Early GC and Advanced GC**

|  | Healthy | Early GC | Advanced GC | p |
| --- | --- | --- | --- | --- |
| N | 61 | 48 | 30 | — |
| Age | 51.61±11.68 | 67.17±9.91 | 69.27±8.06 | 0.000 |
| BMI (kg/m^2^) | 23.96±3.3 | 22.39±3.51 | 21.96±2.99 | 0.013 |
| CA125 | 11.66±5.79 | 22.76±39 | 14.36±7.04 | 0.140 |
| CA153 | 10.73±4.5 | 9.15±4.21 | 8.22±6.16 | 0.244 |
| CA724 | 3.13±2.87 | 4.01±5.55 | 2.68±2.61 | 0.391 |
| CA199 | 10.36±15.15 | 417.73±2332.12 | 213.8±678.11 | 0.427 |
| CEA | 1.98±1.21 | 4.4±4.52 | 12.4±32.96 | 0.022 |
| AFP | 2.78±1.06 | 2.78±1.68 | 171.75±918.54 | 0.247 |
| Male | 37 | 38 | 20 | 0.116 |
| Female | 24 | 10 | 10 |  |
| Smoking history | 16 | 14 | 8 | 0.939 |
| Drinking history | 17 | 23 | 10 | 0.091 |
| Hypertension history | 14 | 19 | 12 | 0.097 |
| Diabetes history | 5 | 2 | 2 | 0.696 |
