# Supplementary material for: Analysis of bacterial diversity and community structure in gastric juice of patients with advanced gastric cancer
Source: Discov Oncol. 2023 Jan 20;14:7. doi: 10.1007/s12672-023-00612-7 (PMC9860007; doi:10.1007/s12672-023-00612-7)
Supplement: Supplementary file 4 — Additional file 4: Table S4. Table of diversity indices about Healthy group, Early GC and Advanced GC. The indexes of community richness were chao and ace. The indexes of community diversity were shannon, simpson and coverage. [file 12672_2023_612_MOESM4_ESM.docx]

**Additional file 4 Table 4. Table of diversity indices about Healthy group, Early GC and Advanced GC. The indexes of community richness were chao and ace. The indexes of community diversity were shannon, simpson and coverage.**

| **Groups** | **shannon** | **simpson** | **ace** | **chao** | **coverage** |
| --- | --- | --- | --- | --- | --- |
| Healthy Group | 4.612 | 0.024 | 568.332 | 565.500 | 0.998 |
|  | 4.982 | 0.015 | 388.229 | 394.375 | 1.000 |
|  | 2.323 | 0.148 | 97.283 | 98.143 | 1.000 |
|  | 5.034 | 0.014 | 435.484 | 437.583 | 1.000 |
|  | 4.908 | 0.017 | 439.349 | 442.059 | 0.999 |
|  | 2.599 | 0.130 | 104.558 | 106.500 | 1.000 |
|  | 4.782 | 0.022 | 636.783 | 639.000 | 0.998 |
|  | 4.669 | 0.028 | 492.044 | 492.000 | 0.999 |
|  | 2.446 | 0.143 | 116.961 | 126.000 | 0.999 |
|  | 2.377 | 0.138 | 86.036 | 85.000 | 1.000 |
|  | 4.704 | 0.025 | 516.253 | 516.885 | 1.000 |
|  | 3.127 | 0.142 | 580.714 | 583.552 | 0.997 |
|  | 4.824 | 0.018 | 565.570 | 577.750 | 0.999 |
|  | 2.482 | 0.160 | 416.334 | 396.382 | 0.998 |
|  | 4.080 | 0.040 | 437.465 | 440.000 | 0.999 |
|  | 2.684 | 0.130 | 99.914 | 98.111 | 1.000 |
|  | 1.338 | 0.597 | 503.487 | 486.339 | 0.996 |
|  | 1.798 | 0.344 | 86.128 | 83.750 | 0.999 |
|  | 4.093 | 0.043 | 467.466 | 471.677 | 0.999 |
|  | 3.802 | 0.073 | 554.290 | 557.788 | 0.998 |
|  | 1.734 | 0.397 | 81.863 | 81.875 | 1.000 |
|  | 2.455 | 0.121 | 366.738 | 387.833 | 0.998 |
|  | 2.649 | 0.131 | 131.883 | 132.000 | 0.999 |
|  | 2.399 | 0.137 | 75.732 | 86.000 | 1.000 |
|  | 1.315 | 0.419 | 337.305 | 324.438 | 0.998 |
|  | 2.160 | 0.266 | 127.031 | 107.000 | 1.000 |
|  | 1.038 | 0.434 | 104.849 | 86.200 | 0.999 |
|  | 0.948 | 0.449 | 109.765 | 76.667 | 0.999 |
|  | 2.288 | 0.184 | 131.259 | 129.083 | 0.999 |
|  | 2.069 | 0.273 | 126.393 | 134.857 | 1.000 |
|  | 2.383 | 0.161 | 114.003 | 101.667 | 1.000 |
|  | 2.816 | 0.093 | 122.148 | 130.000 | 1.000 |
|  | 2.915 | 0.103 | 138.400 | 139.300 | 1.000 |
|  | 1.233 | 0.442 | 234.566 | 229.625 | 0.999 |
|  | 2.340 | 0.207 | 132.767 | 128.615 | 1.000 |
|  | 2.520 | 0.147 | 236.052 | 220.111 | 0.999 |
|  | 2.857 | 0.096 | 220.894 | 205.552 | 0.999 |
|  | 2.743 | 0.100 | 124.106 | 126.125 | 1.000 |
|  | 1.076 | 0.500 | 116.037 | 112.500 | 1.000 |
|  | 1.071 | 0.509 | 118.500 | 122.000 | 0.999 |
|  | 1.148 | 0.456 | 125.766 | 126.750 | 0.999 |
|  | 2.944 | 0.091 | 180.578 | 192.500 | 0.999 |
|  | 1.214 | 0.430 | 115.539 | 115.875 | 1.000 |
|  | 2.495 | 0.167 | 167.717 | 172.077 | 0.999 |
|  | 1.772 | 0.300 | 134.379 | 129.500 | 1.000 |
|  | 2.534 | 0.132 | 142.168 | 139.867 | 1.000 |
|  | 1.060 | 0.500 | 160.135 | 148.455 | 0.999 |
|  | 2.139 | 0.206 | 148.394 | 153.071 | 0.999 |
|  | 1.059 | 0.490 | 136.184 | 129.545 | 0.999 |
|  | 1.081 | 0.469 | 117.144 | 107.474 | 0.999 |
|  | 0.935 | 0.527 | 59.177 | 54.667 | 1.000 |
|  | 0.968 | 0.525 | 112.956 | 113.429 | 0.999 |
|  | 0.945 | 0.545 | 128.833 | 127.071 | 0.999 |
|  | 1.851 | 0.300 | 165.288 | 168.167 | 0.999 |
|  | 0.874 | 0.672 | 142.538 | 148.769 | 0.999 |
|  | 1.155 | 0.473 | 119.851 | 116.667 | 1.000 |
|  | 1.111 | 0.459 | 153.791 | 159.176 | 0.999 |
|  | 0.997 | 0.515 | 99.327 | 94.400 | 0.999 |
|  | 2.817 | 0.096 | 107.649 | 107.000 | 1.000 |
|  | 2.706 | 0.114 | 172.072 | 170.000 | 0.999 |
|  | 0.988 | 0.529 | 79.437 | 75.077 | 1.000 |
| Early GC Group | 2.354 | 0.187 | 311.263 | 247.182 | 0.998 |
|  | 4.226 | 0.054 | 346.982 | 346.571 | 1.000 |
|  | 1.626 | 0.300 | 89.139 | 77.100 | 1.000 |
|  | 1.535 | 0.308 | 88.587 | 81.583 | 1.000 |
|  | 2.260 | 0.177 | 112.516 | 137.250 | 1.000 |
|  | 1.661 | 0.328 | 380.583 | 303.129 | 0.998 |
|  | 1.517 | 0.381 | 85.772 | 87.000 | 1.000 |
|  | 1.777 | 0.431 | 331.556 | 261.048 | 0.999 |
|  | 2.181 | 0.304 | 457.980 | 453.625 | 0.998 |
|  | 5.180 | 0.013 | 644.414 | 647.194 | 0.999 |
|  | 1.958 | 0.315 | 292.882 | 222.875 | 0.999 |
|  | 2.127 | 0.222 | 90.748 | 90.500 | 1.000 |
|  | 2.327 | 0.188 | 146.234 | 170.000 | 0.999 |
|  | 2.360 | 0.151 | 91.582 | 98.000 | 1.000 |
|  | 2.565 | 0.141 | 124.618 | 125.600 | 1.000 |
|  | 2.555 | 0.199 | 157.271 | 157.571 | 1.000 |
|  | 2.741 | 0.118 | 120.451 | 120.500 | 1.000 |
|  | 2.900 | 0.158 | 248.426 | 246.577 | 0.999 |
|  | 1.555 | 0.353 | 125.055 | 125.273 | 1.000 |
|  | 2.628 | 0.103 | 80.630 | 79.000 | 1.000 |
|  | 2.494 | 0.182 | 217.147 | 166.300 | 0.999 |
|  | 2.038 | 0.330 | 151.679 | 155.364 | 0.999 |
|  | 1.912 | 0.312 | 152.796 | 136.100 | 0.999 |
|  | 1.816 | 0.312 | 100.645 | 99.111 | 1.000 |
|  | 2.312 | 0.173 | 107.835 | 104.571 | 1.000 |
|  | 1.334 | 0.518 | 113.950 | 114.077 | 0.999 |
|  | 2.360 | 0.199 | 101.708 | 100.143 | 1.000 |
|  | 0.611 | 0.795 | 86.089 | 84.000 | 1.000 |
|  | 1.882 | 0.389 | 149.344 | 146.667 | 1.000 |
|  | 2.258 | 0.222 | 149.347 | 134.000 | 0.999 |
|  | 1.657 | 0.411 | 124.356 | 120.500 | 0.999 |
|  | 2.293 | 0.185 | 141.860 | 148.273 | 1.000 |
|  | 1.796 | 0.300 | 85.620 | 84.750 | 1.000 |
|  | 1.557 | 0.354 | 64.521 | 66.000 | 1.000 |
|  | 2.392 | 0.157 | 114.463 | 117.125 | 1.000 |
|  | 1.941 | 0.260 | 97.246 | 93.091 | 1.000 |
|  | 1.492 | 0.333 | 94.050 | 81.000 | 1.000 |
|  | 1.812 | 0.377 | 126.440 | 123.100 | 1.000 |
|  | 2.244 | 0.271 | 132.310 | 133.250 | 1.000 |
|  | 2.378 | 0.195 | 91.199 | 89.000 | 1.000 |
|  | 2.147 | 0.226 | 135.549 | 129.647 | 0.999 |
|  | 1.491 | 0.502 | 95.901 | 94.750 | 1.000 |
|  | 2.053 | 0.209 | 89.668 | 87.000 | 1.000 |
|  | 1.247 | 0.442 | 99.719 | 105.000 | 1.000 |
|  | 1.047 | 0.506 | 66.606 | 64.333 | 1.000 |
|  | 2.879 | 0.106 | 116.624 | 116.875 | 1.000 |
|  | 2.421 | 0.134 | 123.792 | 120.000 | 0.999 |
|  | 2.292 | 0.202 | 90.644 | 95.200 | 1.000 |
| Advanced GC Group | 2.547 | 0.166 | 335.677 | 345.294 | 0.999 |
|  | 2.687 | 0.099 | 208.068 | 200.138 | 0.999 |
|  | 2.671 | 0.113 | 195.480 | 196.429 | 0.999 |
|  | 1.791 | 0.259 | 121.933 | 117.167 | 0.999 |
|  | 1.957 | 0.300 | 100.421 | 96.100 | 1.000 |
|  | 1.918 | 0.309 | 362.281 | 295.731 | 0.998 |
|  | 2.048 | 0.303 | 440.558 | 428.159 | 0.997 |
|  | 2.223 | 0.250 | 108.194 | 119.200 | 0.999 |
|  | 2.168 | 0.153 | 58.761 | 56.200 | 1.000 |
|  | 1.836 | 0.306 | 161.827 | 146.000 | 0.999 |
|  | 1.764 | 0.371 | 70.804 | 77.000 | 1.000 |
|  | 2.588 | 0.141 | 379.084 | 367.109 | 0.998 |
|  | 1.713 | 0.258 | 70.942 | 65.333 | 1.000 |
|  | 2.906 | 0.110 | 120.477 | 118.600 | 1.000 |
|  | 2.445 | 0.121 | 161.308 | 106.667 | 1.000 |
|  | 0.174 | 0.954 | 35.823 | 36.333 | 1.000 |
|  | 2.657 | 0.115 | 105.810 | 101.600 | 1.000 |
|  | 2.669 | 0.151 | 132.942 | 130.800 | 1.000 |
|  | 2.611 | 0.128 | 110.546 | 111.000 | 1.000 |
|  | 2.565 | 0.130 | 128.177 | 137.333 | 0.999 |
|  | 1.321 | 0.541 | 141.941 | 156.625 | 0.999 |
|  | 2.893 | 0.082 | 92.910 | 101.000 | 1.000 |
|  | 2.680 | 0.130 | 128.603 | 155.000 | 1.000 |
|  | 2.282 | 0.182 | 96.341 | 101.500 | 1.000 |
|  | 2.624 | 0.175 | 158.103 | 168.500 | 1.000 |
|  | 2.450 | 0.193 | 142.927 | 157.500 | 0.999 |
|  | 1.697 | 0.247 | 76.849 | 74.600 | 1.000 |
|  | 1.758 | 0.299 | 119.299 | 89.600 | 1.000 |
|  | 1.723 | 0.370 | 87.777 | 122.500 | 1.000 |
|  | 2.339 | 0.146 | 90.743 | 90.000 | 1.000 |
